# The Effect of Tranexamic Acid on Estimated Blood Loss and Transfusion Rates in Children with Cerebral Palsy Undergoing Single-Event Multi-Level Surgery, a Retrospective Study

**DOI:** 10.3390/children12030267

**Published:** 2025-02-21

**Authors:** Florence Julien-Marsollier, Anne-Laure Simon, Pierre Pardessus, Ana Presedo, Brice Ilharreborde, Souhayl Dahmani

**Affiliations:** 1 Department of Anesthesia and Intensive Care, Robert Debré Hospital, 48 Boulevard Sérurier, 75019 Paris, France; pierre.pardessus@aphp.fr (P.P.); souhayl.dahmani@aphp.fr (S.D.); 2 Department of Pediatric Orthopedic Surgery, Université de Paris Cité, 85 Boulevard Saint Germain, 75006 Paris, France; anne-laure.simon@aphp.fr (A.-L.S.); ana.presedo@aphp.fr (A.P.); brice.ilharreborde@aphp.fr (B.I.); 3 DMU Parabol, Université APHP Nord Université Paris Cité, 85 Boulevard Saint Germain, 75006 Paris, France; 4 Department of Pediatric Orthopaedic, Robert Debré University Hospital, 75019 Paris, France

**Keywords:** cerebral palsy, blood loss prevention, bone surgery, early rehabilitation

## Abstract

*Purpose:* Multiple osteotomies are frequently associated with single-event multi-level surgery (SEMLS) in children with cerebral palsy (CP). However, appropriate management of perioperative blood loss is crucial for decreasing the length of hospital stay and early rehabilitation. Tranexamic acid (TxA) has been proven to significantly reduce perioperative bleeding in multiple major orthopedic surgeries. The aim of this study was to investigate the effectiveness of TxA in decreasing blood loss in children with CP undergoing SEMLS procedures. *Materials and Methods:* Between September 2016 and September 2022, 101 consecutive children with CP who underwent SEMLS were identified—50 patients did not receive TxA peri-operatively (Control Group, from September 2016 to September 2018), and 51 patients received TxA (TxA Group since September 2018). Bleeding, hemoglobin levels, transfusion rate, length of hospital stay and postoperative hematocrit were compared between the groups. The predictive factors of blood transfusion were determined. *Results:* The transfusion rate significantly decreased in the TxA Group (43.3% vs. 4%, *p* < 0.001). The use of TxA and epidural analgesia were the identified factors for a significant transfusion rate decrease. Bleeding (estimated red cell loss) was decreased in the TxA group in comparison to the control group 22.3 [17.3–27.3] versus 33.78 [27.4–40.2], *p* < 0.05). The hospital length of stay significantly decreased in the TxA group (7.3 vs. 6 days, *p* = 0.01). No TxA-related complications occurred in any of the patients. *Conclusions:* A blood loss prevention strategy based on a low dose of TxA in children scheduled for SEMLS significantly decreased bleeding and transfusion rates, allowing an earlier discharge from the hospital for patients.

## 1. Introduction

Cerebral palsy (CP) is a neurodevelopmental disease characterized by an association of abnormalities of muscle tone, movement and motor skills secondary to a hypoxic injury in the developing brain; its prevalence is high in developed countries (2–3 per 1000 live births) [1,2]. This prevalence increases (40–100 among 1000 births) in children born below 28 weeks of gestation, with different risk factors, such as perinatal infection, intrauterine growth restriction and use of preterm antibiotics, all associated with CP [3]. Given the major improvements in the management of preterm infants and the subsequent decrease in their mortality, the prevalence of CP remains unchanged or even increased. A high proportion of patients (68%) with hypoxic injuries develop hypertonic muscles with a spasticity state that affects gait and movements but, more importantly, muscular imbalance responsible for musculo-tendinous and osseous deformities.

Therefore, the management of patients with CP usually involves physiotherapy, orthoprosthesis and subsequent surgical corrections of these deformities. Single-event multi-level surgery (SEMLS), defined by clinical gait analysis (CGA), is used to treat lower-limb skeletal deformities and improve walking disorders in children with CP [4]. This surgery is associated with major bleeding, especially when osteotomies are performed. Consequently, usual blood-saving strategies used in many other surgeries appear relevant to the current situation.

During the last few decades, blood-saving strategies have been developed to decrease blood transfusion during major orthopedic surgeries, such as joint replacement in adults and spine surgeries in children [5]. Among those strategies, tranexamic acid (TxA) has been largely evidenced in adults during cardiac surgery [6], trauma [7], orthopedic surgery [8], obstetrics [9] and neurosurgery [10]. In children, similar evidence in favor of TxA use has emerged from studies in cardiac surgery [11], trauma [11], scoliosis surgery [5] and neurosurgery [12]. However, two recent studies investigating the effectiveness of TxA in children with CP scheduled for orthopedic surgery found contradictory results. The first found a decrease in the bleeding, while the second observed no difference [13,14].

In the present study, we evaluated the effectiveness of tranexamic acid on patients with cerebral palsy who are undergoing single-stage multi-level osteotomies. To decrease the bleeding in the perioperative time. The primary outcome of the study was the proportion of patients with CP who received a perioperative allogenic blood transfusion. Secondary outcomes were (1) factors associated with TxA-related decrease in transfusion, (2) the effect of this strategy on perioperative blood loss and (3) hospital length of stay.

## 2. Materials and Methods

### 2.1. Ethics

The study was approved by the local ethics (Comité d’évaluation de l’éthique de la recherche de l’hôpital Robert Debré, Chairman Dr Sophie Crepon, N°138) and by the national committee for electronic data recording (CNIL).

### 2.2. Inclusion and Exclusion Criteria

Inclusion criteria were all children with CP undergoing an SEMLS with osteotomies in the defined time periods. Exclusion criteria were any contraindication to TxA administration (uncontrolled epilepsy or preoperative hypercoagulable abnormalities) and patient files with missing data.

### 2.3. Design of This Study

This study consists of retrospective data collection and analysis from the hospital medical records. The use of TxA was introduced in the current institution in September 2018. It consisted of administration of TxA in the intraoperative period with a loading dose (10 mg·kg^−1^), followed by a continuous infusion (5 mg·kg·h^−1^) until the end of the procedure. Consequently, two sets of patients were compared: the control group (no TxA) before 2018 and the intervention group (TxA) after 2018.

### 2.4. Perioperative Anesthesia

Anesthesia protocols were standardized in both groups according to institutional and local protocols during the two study periods. Anesthesia was performed with a volatile induction using sevoflurane, sufentanil (0.2 µg·kg^−1^) and a muscle relaxant (atracurium 0.5 mg·kg^−1^). Anesthesia was maintained with sevoflurane (one Minimal Alveolar Concentration (MAC) adjusted for age in a mixture of O_2_/N_2_O 50%/50%). An epidural catheter was inserted before the beginning of surgery with a continuous infusion of ropivacaine for intraoperative and postoperative analgesia (ropivacaine 1%, infusion rate 0.2 mL/h). Intraoperative sufentanil boluses were administered during surgery in order to maintain mean arterial pressure and heart rate within 20% of the preoperative values. All patients in both groups received dexamethasone at the beginning of the surgery (0.15 mg·kg^−1^ after induction of anesthesia). Body temperature was controlled during the whole procedure and maintained between 36.5 °C and 37 °C. Muscle relaxant was infused continuously during surgery and reversed at the end of the procedure. Intraoperative fluid management consisted of Ringer’s Lactate administered according to the Holliday and Segar formulae [15].

Postoperative multimodal analgesia was standardized and included paracetamol, NSAIDs (ketoprofen) and epidural continuous infusion for 3 days. Nalbuphine was administered as breakthrough analgesia according to a visual analogue pain scale adapted to the age of the patient.

Hemoglobin levels were systematically checked on the day of the surgery and on postoperative days 1 and 3. The hemoglobin rate requiring a blood transfusion was the same during the whole period, among national recommendations (7 g/dL or 10 g/dL if the patients had a cardiac comorbidity).

### 2.5. Operative Procedure

All surgeries were performed by one or two pediatric orthopedic senior surgeons and fellows specialized in neuromuscular orthopedics.

### 2.6. Data Collection

**Figure 1:**
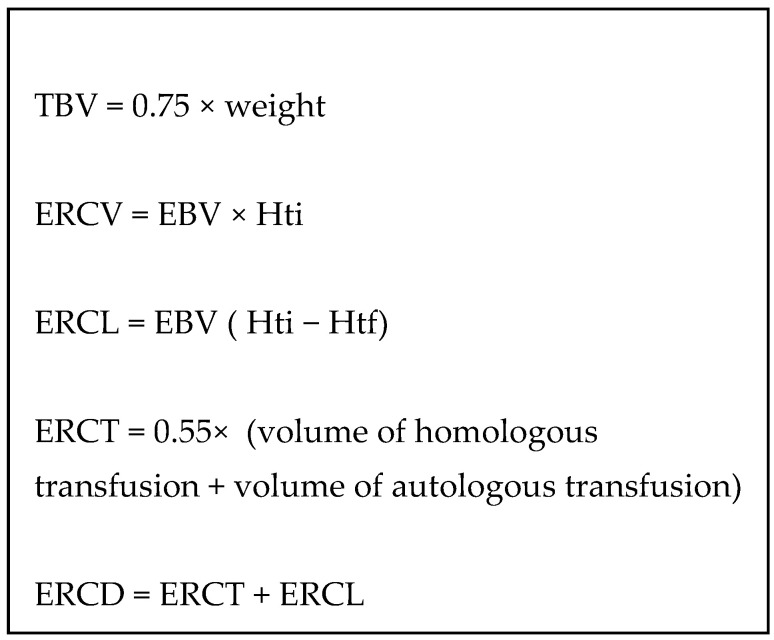
Derived parameters with Cell-saver used.

Data collected consisted of demographic data (age, weight), type of surgery (number of osteotomies or musculotendinous lengthenings), ASA grade (classification used in anesthesia to evaluate the comorbidities of the patients), use of epidural analgesia, hemoglobin levels (preoperative day −1 and at postoperative days 1 and 3), duration of surgery, duration of anesthesia, TxA use, intraoperative and perioperative blood transfusions, and length of hospital stays. Using the weight-related total blood volume formulae previously published, the red cell deficit was estimated, including autologous or heterologous blood transfusion, when required during the perioperative period [16]. Thus, the estimated red cell loss (ERCV) is an estimation of the bleeding based on the weight and the variation of hemoglobin rate. The estimated red cell deficit (ERCL) calculated between two time points corresponded to the product of total blood volume (TBV = 0.75 × weight for an adolescent) and the change in hematocrit between these two measurements. Red blood cell volume (ERCV) corresponded to the product of TBV and hematocrit. The volume of red blood cells transfused (ERCT) combined autologous transfusion (intraoperative Cell-Saver) and heterologous transfusion, with a weighting factor of 0.55 linked to the estimated quantity of red blood cells in a packed red blood cell. Thus, the addition of the red blood cell deficit (ERCL) and the volume of red blood cells transfused (ERCT) corresponded to perioperative bleeding (ERCD) (Figure 1).

### 2.7. Statistical Analysis

Power calculation was based on a previously published study exploring the use of a similar protocol (preoperative hemoglobin optimization and TxA use) [17]. Based on the rate of transfusion observed in 20 patients in the control group (45%) and expecting a reduction of 6 times the rate of transfusion (77/13), the estimated sample size in each group was 50 with an alpha value of 0.05 and a power analysis of 90% [18].

Descriptive statistics were reported using median and range for continuous variables and percentages (%) for discrete ones. Statistical comparisons between the two cohorts used the Mann-Whitney non-parametric tests for continuous variables and the Chi-squared test. (or Fisher’s exact test) for discrete variables. After the normality test, non-parametric tests were performed.

In order to determine factors associated with TxA-related decrease in blood transfusion, a univariate analysis was performed for factors that might impact perioperative transfusion, namely: TxA use [12], type of surgery [18], duration of surgery [19] and use of epidural anesthesia [20]. All factors exhibiting a *p*-value < 0.2 were entered in a stepwise multivariable analysis logistic regression.

Statistical analysis was carried out on SPSS 26.0 (IBM Corp. Released 2019. IBM SPSS Statistics for Windows, Version 26.0. IBM Corp., Armonk, NY, USA). The level of significance was set at *p* < 0.05.

## 3. Results

Fifty-three consecutive patients were eligible in the control group and 52 consecutive ones in the TxA group. Four patients were excluded: three in the control group (missing data) and one in the TxA group because blood saving-strategies were not used. Required data were complete in all other patients. The two cohorts were described in Table 1.

Concerning the primary outcome of the study, two patients (4%) were transfused in the TxA group and 22 (43,3%) in the control group (*p* < 0.001). Bleeding (estimated red cell loss in mL·Kg^−1^) was decreased in the TxA group in comparison to the control group 22.3 [17.3–27.3] versus 33.78 [27.4–40.2], *p* < 0.05). The hospital length of stay was significantly reduced in the TxA group 6 [5,6] versus 7.3 [7,8], *p* = 0.01) (Table 2 and Table 3).

Regarding factors associated with an increased risk of blood transfusion, the multivariable analysis found the use of TxA and epidural analgesia as protective, while an increased duration of surgery was associated with an increased risk of blood transfusion (Table 4). No thromboembolic complications were observed.

## 4. Discussion

In the current study, the use of intraoperative TxA in children with CP scheduled for SEMLS significantly decreased the transfusion rate in the perioperative period. Results also found this treatment to decrease total blood loss and hospital length of stay.

The implementation of a blood-saving strategy for hemorrhagic surgery is widely used in current surgical practice. The use of TxA is largely accepted in cardiac surgery, scoliosis or major trauma cases [9]. In pediatrics, recent studies have shown that during craniosynostosis surgery and scoliosis, the use of TxA decreases bleeding [10,12]. However, a recent study during femoral derotation in children with CP did not find a significant reduction in blood transfusion associated with the use of TxA probably [18]. In contrast, another study performed on the same population and for the same surgery found a decrease in blood transfusion [14]. The difference can be explained by the closed size of the clot and the missing data in the retrospective study.

### 4.1. The Low-Dose TxA Protocol

The effective dose of TxA to be administered intraoperatively to decrease bleeding and blood transfusion remains a subject of discussion. A high dose (loading dose of 50 mg·kg^−1^ then an infusion of 10 mg·Kg^−1^·h^−1^) and a low dose (loading dose of 10 mg/kg then an infusion of 5 mg·Kg^−1^·h^−1^) are the procedures described in the literature. However, a recent study on pediatric craniostenosis found no significant difference between these two protocols [17]. During spine surgery, studies found a statistical correlation between the dose of TxA and the reduction of blood transfusion [21]. Based on TXA pharmacokinetics, Goobie and Faraoni recommend a dosing regimen of between 10 to 30 mg/kg loading dose followed by 5 to 10 mg/kg/h maintenance infusion rate for pediatric trauma and surgery [22]. Moreover, the authors of both studies suggested using a minimal dose of TxA in order to avoid potential side effects, particularly venous thrombosis. Given the high prevalence of epilepsy and thrombotic risks in the studied population, a low dose of TxA was used in the current study. Interestingly, no thromboembolic complications were observed, consistent with previous literature findings [18]. However, our study was not able to investigate these side effects, and no conclusion can be made on this specific topic.

### 4.2. Protective and Risk Factors for Blood Transfusion

The rate of blood transfusion in our control group was higher in comparison to previous studies on the same topic: 43% in our study versus 36% [23]. This may be related to differences in surgical techniques (lower-limb osteotomies and hip reconstructions). This is consistent with the difference between transfused and non-transfused patients (Table 3). The number of osteotomy levels and duration of surgery were independent predictors of blood transfusion in our study. Alternatively, differences in intraoperative fluid management practices could also explain this result. The rate of blood transfusion was decreased in the TxA group despite the higher number of multi-level osteotomy surgeries. Moreover, the multivariable analysis still demonstrated TxA to be a protective factor, even when adjusting for other risk factors of bleeding and transfusion.

Epidural analgesia was found to be protective against blood transfusion. Although this result cannot directly explain the current study findings, one can hypothesize that the decrease in venous return may have decreased venous blood flow and subsequent loss. Such a mechanism has been advocated for explaining the effects of intrathecal morphine on blood loss during spine surgery in children [24].

We chose the rate of transfusion by day 1 post-surgery in order to decrease the bias linked to heterogeneity in perioperative fluid management related to anesthetists’ experience [25]. This was particularly important given that no hemodynamic monitoring was used to adapt the vascular filling to hemodynamic conditions.

### 4.3. Blood Saving Procedures for Early Rehabilitation

Fast-track rehabilitation is a promising practice that optimizes patient management [26]. This strategy is increasingly used for different types of surgery [27]. In our study, the hospital length of stay was reduced by 1 day in CP children undergoing SEMLS with the TxA use. This earlier discharge from the hospital could potentially allow improved utilization of these accelerated rehabilitation protocols after surgery by allowing a better tolerance of surgical stress. However, further additional research will be needed to investigate these associations.

### 4.4. Limitations

Our study has some limitations. Firstly, this is not a randomized trial, and consequently, many biases may have occurred. Secondly, this is a monocentric study, and results may vary according to surgical techniques and procedures. However, given the same senior surgeons were involved in both the control and TxA groups, the surgical technique was standardized between our two groups, further highlighting the benefit of perioperative TxA in SEMLS. Estimated blood loss and deficit were calculated based on the difference in the hematocrit between the day before surgery and day 1, as described in a previous study [16]. This represents an estimation rather than real blood loss. The difference between the two groups of patients in terms of surgery and duration might have biased results on transfusion. However, this could have negatively impacted TxA efficacy. In addition, the multivariable analysis found TxA to remain protective even when adjusting for confounding factors.

In conclusion, the current study found that the systematic use of a blood saving strategy based on the use of low dose tranexamic acid in children scheduled for SEMLS is associated with a decrease in the transfusion rate and blood loss, and allows an earlier discharge from hospital for the patients.

## Figures and Tables

**Table 1:** Descriptive data. Results are expressed as median [ranges] or N (%).

	Control Group N = 50	TxA Group N = 51	*p* Value
Age (years)	10.75 [4.2–17.8]	9.2 [1.8–14.6]	0.114
Weight (Kg)	27 [13.5–52]	24 [9–50]	0.131
ASA			
1	1 (3.3)	2 (6.7)	
2	22 (73.3)	21 (70)	
3	7 (23.3)	7 (23.3)	
Hemoglobin day before surgery	13.1 [11.2–15.1]	13.2 [9.1–15.1]	0.636
Duration of anesthesia (h)	4.5 [3–7.5]	4.6 [2.5–5]	0.192
Duration of surgery (h)	3 [2–6.5]	3 [1.5–4]	0.280
Perioperative epidural analgesia	43 (83.3)	46 (90.1)	0.373
Proximal Femoral osteotomie	32 (64)	34 (66)	0.232
Distal femoral osteotomie	22 (44)	24 (47)	0.187
Tibial osteotomie	22 (44)	24 (47)	0.187
Pelvic osteotomie	18 (60)	19 (37)	0.254

**Table 2:** Univariate analysis results are expressed as median [ranges] or N (%).

	Control Group N = 50	TxA Group N = 51	*p* Value
Hemogobin at postoperative day 1 (g/dL)	9.2 [6.4–13.1]	9.5 [7–13.1]	0.129
Hemoglobin at postoperative day 3 (g/dL)	8.9 [5.6–14.2]	10.6 [8.2–13.3]	0.011
Intraoperatrive Transfusion	**3 (10)**	**0 (0)**	**0.001**
**Postoperative Transfusion**	**13 (43.3)**	**1 (3.3)**	**<0.001**
**Fluid intake (L**·**Kg^−1^)**	**1.5 [0.5–4]**	**1 [0.4–3.5]**	**0.015**
**Hospital length (day)**	**7 [6,7,8]**	**6 [6–7]**	**0.001**
**ERCV lost (mL**·**kg^−1^) at day 3**	**33.78 [27.37–40.2]**	**22.33 [17.33–27.34]**	**0.005**

**Table 3:** Univariate analysis results are expressed as median [ranges] or N among the type of osteotomies.

	**Control Group**	**TxA Group**	***p* Value**
**Proximal Femoral osteotomie**	**N = 32**	**N = 34**	
Hemogobin at postoperative day 1 (g/dL)	8.6 [6.9–12.1]	9.5 [7–12.9]	0.03
Hemoglobin at postoperative day 3 (g/dL)	8.2 [6.6–14.2]	9.6 [8.6–13.2]	0.02
Intraoperative Transfusion	1	0	0.04
Postoperative Transfusion	6	0	<0.001
**Distal femoral osteotomie**	**N = 22**	**N = 24**	
Hemogobin at postoperative day 1 (g/dL)	9.0 [6.4–13.0]	9.5 [7–13.1]	0.19
Hemoglobin at postoperative day 3 (g/dL)	8.6 [5.6–14.2]	9.2 [8.2–12.9]	0.01
Intraoperative Transfusion	0	0	0.001
Postoperative Transfusion	0	0	<0.02
**Pelvic osteotomie**	**N = 18**	**N = 19**	
Hemogobin at postoperative day 1 (g/dL)	8.4 [6.4–11.8]	9.1 [7–12.9]	**0.001**
Hemoglobin at postoperative day 3 (g/dL)	8.0 [5.6–11.2]	10.4 [8.1–13.0]	**0.04**
Intraoperative Transfusion	2	0	<0.02
Postoperative Transfusion	7	1	<0.001

**Table 4:** Multivariate analysis for blood transfusion.

	Univariate			Multivariable	
	No Transfusion (N, %)	Transfusion (N, %)	*p* Value	OR	95% Confidence Interval of OR
TxA	35 (47.9)	1 (1.4)	<0.001	0.028	[0.002–0.38]
Epidural analgesia	50 (68.5)	11 (83.6)	0.07	0.082	[0.01–0.681]
Length of surgery (h)	3 [1.5–4]	3.25 [2–6.5]	0.15	3.1	[1.2–7.6]

## Data Availability

The original contributions presented in this study are included in the article. Further inquiries can be directed to the corresponding author.
